# Magnetoluminescence from trion and biexciton in type-II quantum dot

**DOI:** 10.1186/1556-276X-6-351

**Published:** 2011-04-20

**Authors:** Rin Okuyama, Mikio Eto, Hiroyuki Hyuga

**Affiliations:** 1 Faculty of Science and Technology, Keio University, 3-14-1 Hiyoshi, Kohoku-ku, Yokohama 223-8522, Japan

## Abstract

We theoretically investigate optical Aharonov-Bohm (AB) effects on trion and biexciton in the type-II semiconductor quantum dots, in which holes are localized near the center of the dot, and electrons are confined in a ring structure formed around the dot. Many-particle states are calculated numerically by the exact diagonalization method. Two electrons in trion and biexciton are strongly correlated to each other, forming a Wigner molecule. Since the relative motion of electrons are frozen, the Wigner molecule behaves as a composite particle whose mass and charges are twice those of an electron. As a result, the period of AB oscillation for trion and biexciton becomes *h*/2*e *as a function of magnetic flux penetrating the ring. We find that the magnetoluminescence spectra from trion and biexciton change discontinuously as the magnetic flux increases by *h*/2*e*.

PACS: 71.35.Ji, 73.21.-b, 73.21.La, 78.67.Hc

## Introduction

Rapid advance in nanotechnology has allowed us to fabricate ring structures whose circumference is shorter than the phase coherent length. In these systems, the persistent current induced by the Aharonov-Bohm (AB) effect was predicted theoretically [1], and observed both for metallic rings in the diffusive regime and semiconductor rings in the ballistic regime [2,3]. In the semiconductor rings, the theory well explains the experimental results. In the metallic rings, however, the observed current was much larger than the theoretical prediction. This should be ascribed to the electron-electron interaction in the rings, which has not been fully understood.

**Figure 1:**
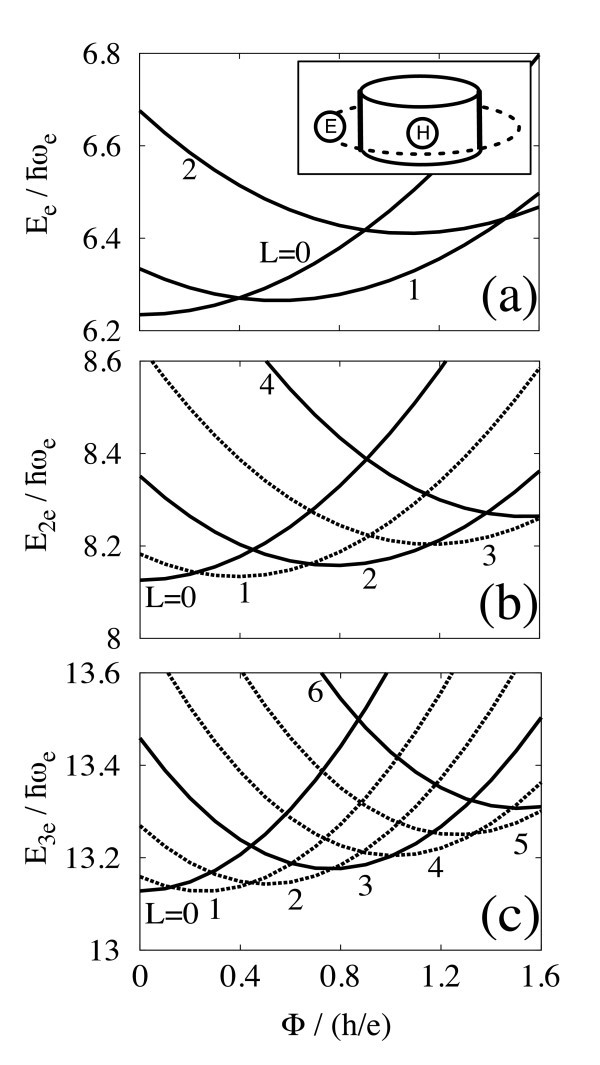
**Low-lying energies for (a) one, (b) two, and (c) three electrons in the type-II quantum dot, as a function of the magnetic flux Φ**. The dot radius *R *equals to the effective Bohr radius *a*_B _= 4*πϵħ*^2^/*m*_e_*e*^2^. Solid and dash lines indicate spin-singlet and triplet, respectively, in (b), whereas they indicate quartet and doublet in (c). The period of AB oscillation becomes *h*/*Ne *for *N *electrons.

In type-II semiconductor quantum dots, such as ZnSeTe and SiGe, holes are localized inside the quantum dots while electrons move in a ring structure formed around the dots (inset in Figure 1a). In a perpendicular magnetic field B, the electrons acquire the AB phase. For the sake of simplicity, suppose that an electron moves in a perfect one-dimensional ring of radius *R*, the Hamiltonian is written as(1)

where  is the angular momentum operator, *m*_e _is the effective mass of electron, and Φ= *π R*^2^*B *is the magnetic flux penetrating the ring. As a result, the angular momentum increases with in the ground state, and the energy oscillates as a function of Φ by the period of *h*/*e *[1]. This AB effect was observed experimentally as the *B *dependence of peak position of luminescence from excitons [4,5], in which the hole motion is almost frozen due to the strong confinement [6]. This is called an optical AB effect.

In this study, we theoretically investigate the correlation effect when more than one electron is put in a type-II quantum dot. First, we calculate the many-electron states in the quasi-one-dimensional ring and find the formation of Wigner molecules [7]. Since the relative motion of electrons is frozen due to the strong correlation, an *N*-electron molecule behaves as a composite particle whose charge and mass are *N *times of those of an electron. In consequence, the energy oscillates with Φ by the period of *h*/*Ne*. This is known as a fractional AB effect [8]. Next, we examine the magnetoluminescence from trion and biexciton in the type-II quantum dot. We show that the peak position and intensity of the luminescence change discontinuously as Φ increases by *h*/2*e*. This indicates the possible observation of Wigner molecules by the optical experiment.

## Model and calculation method

We consider a type-II semiconductor quantum dot formed in a plane. A ring-like potential  is imposed on electrons, while a harmonic potential  on holes. Here, *m*_e _and *m*_h _are the effective masses of electrons and holes, respectively. A magnetic field is applied perpendicularly to the quantum dot.

Parameters *ω*_e_, *ω*_h_, *V*_0_, and *α *are chosen so that *R*, at which *V*_e_(*r*) has the minimum, is eight times larger than the size of hole confinement . The expectation value of the electron radius is approximately *R *in our model.

Using the field operator for electron, , and for hole, , the effective mass Hamiltonian is written as(2)

where *q*_e _= - *e*, *q*_h _= *e*, and ***A***(***r***) is the vector potential; ∇ × ***A ***= - *B***e***_z_*. Note that the exchange interaction between electron and hole is omitted here for the following reason. An electron [hole] wave function Ψ_e_(***r***) [Ψ_h_(***r***)] is written as(3)(4)

where *ψ*_e_(***r***) [*ψ *_h_(***r***)] is an envelope function for an electron [hole] state, and *u*_c_(***r***) [*u*_v_(***r***)] is the Bloch function of the conduction [valence] band edge. *u*_c_(***r***) and *u*_v_(***r***) mainly consist of s- and p-waves, respectively. Since they oscillate in space by the period of the lattice constant, *a*, the exchange interaction between electron and hole is smaller by the order of (*a*/*R*)^2 ^than other terms, e.g., the exchange interaction between two electrons.

The strength of the magnetic field is measured by Φ = *πR*^2^*B*, the flux penetrating the ring of radius *R*. The strength of the Coulomb potential against the kinetic energy increases with *R*/*a*_B_, where *a*_B _= 4*πϵħ*^2^/*m_e_e*^2 ^is the effective Bohr radius. *R*/*a*_B _≳ 1 in the experimental situations [4,5].

The exact diagonalization method is used to take full account of the Coulomb interaction. We calculate the luminescence spectra by the dipole approximation, using obtained energies and wavefunctions of many-body states.

## Results and discussion

### Few electrons without hole

First, we calculate the electronic states in the absence of holes. Figure 1 shows Φ dependence of low-lying energies for (a) one, (b) two, and (c) three electrons confined in *V*_e_(*r*) with *R*/*a*_B _= 1. The total angular momentum *L *is indicated in the figure. For one electron, the angular momentum increases by one in the ground state Φ as increases by about *h*/*e*, and the energy oscillates quasi-periodically with Φ. by the period of *h*/*e*. This suggests that the electronic confinement *V*_e_(*r*) realizes a quasi-one-dimensional electron ring. In contrast to the perfect one-dimensional ring, on the other hand, a diamagnetic shift is seen in our model. As a whole, the energy increases with Φ. This is because the electron radius is shrunk by the magnetic field. For two and three electrons, the angular momentum increases, and the energy oscillates quasi-periodically with Φ in the ground state. The diamagnetic shift is also present. However, the period of AB oscillation becomes about *h*/*Ne *for *N *electrons.

**Figure 2:**
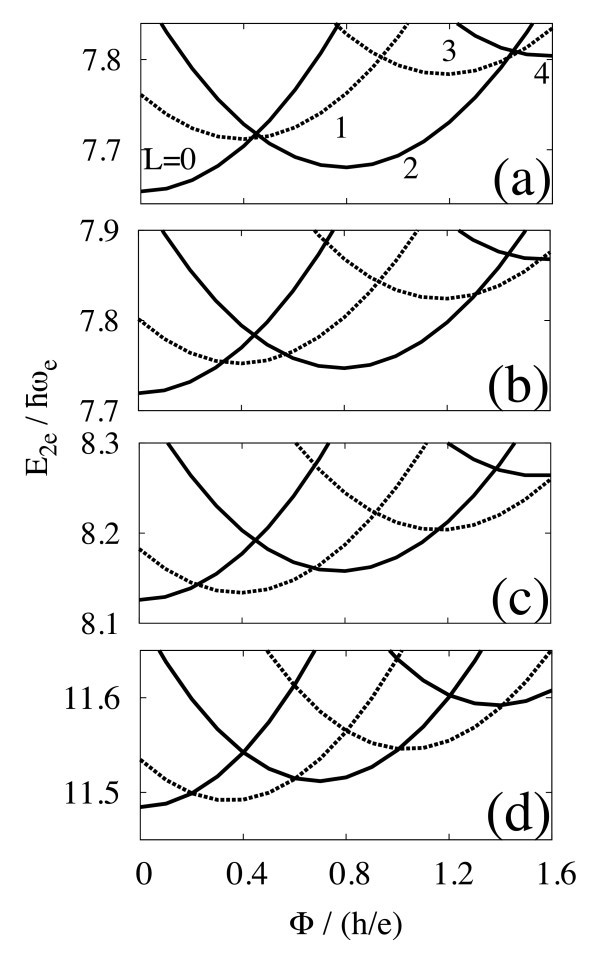
**Low-lying energies for two electrons in the type-II quantum dot, as a function of the magnetic flux Φ**. Solid and dash lines indicate spin-singlet and triplet, respectively. The ratio of the dot radius *R *to the effective Bohr radius *a*_B _= 4*πϵħ*^2^/*m*_e_*e*^2 ^is **(a) **0.01, **(b) **0.1, **(c) **1, and **(d) **10. The ground-state energy oscillates by the period of *h*/2*e *for *R*/*a*_B _≳ 1.

In order to elucidate the relation between the electron-electron interaction and the fractional period of AB oscillation, we examine many-body states for two electrons with changing *R*/*a*_B_. Figure 2 shows low-lying energies with (a) *R *= *a*_B _= 0.01, (b) 0.1, (c) 1, and (d) 10. Without the Coulomb interaction, two electrons occupy the lowest orbital shown in Figure 1a in the ground state. Consequently, the total angular momentum is always even, and the total spin is a singlet. As the strength of the Coulomb interaction increases with *R*/*a*_B_, the exchange interaction lowers energies for spin-triplet states compared to singlet states. For *R*/*a*_B _≳ 1, singlet and triplet states alternatively appear as Φ increases by about *h*/2*e*. Hence, the ground-state energy oscillates with Φ by the period of *h*/2*e*. Note that the period of AB oscillation in the case of *R*/*a*_B _= 10 is slightly shorter than that of *R*/*a*_B _= 1. This is because the Coulomb repulsion between electrons tends to increase the expectation value of the electron radius.

**Figure 3:**
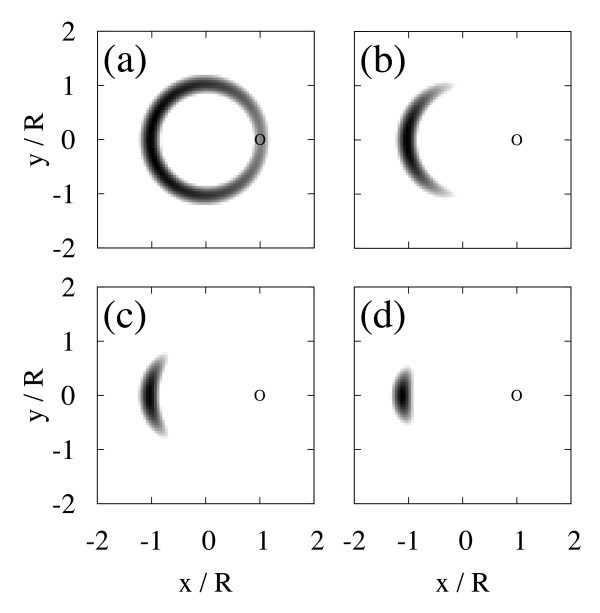
**Gray scale plots of the two-body density for the two-electron ground state at zero magnetic field with (a) *R*/*a*_B _= 0.01, (b) 0.1, (c) 1, and (d) 10**. One electron is fixed at the point indicated by a circle.

We calculate the two-body density(5)

to examine the electric correlation. Figure 3 shows the two-body density for the two-electron ground state at zero magnetic field with (a) *R*/*a*_B _= 0.01, (b) 0.1, (c) 1, and (d) 10. ***r***_0 _is fixed at (*R*, 0), which is indicated by a circle in the plots. For *R*/*a*_B _≳ 1, electrons maximize their distance to be localized at the other side in the ring, that is, a Wigner molecule is formed. Since the relative motion of electrons is frozen, the Wigner molecule behaves as a composite particle whose mass and charge are twice those of an electron. In consequence the ground-state energy oscillates with Φ by the period of about *h*/2*e*.

Similarly, three electrons are localized at apices of an equilateral triangle inscribed in the ring to form a Wigner molecule. The period of AB oscillation in the ground-state energy becomes about *h*/3*e *for *R*/*a*_B _≳ 1.

The total spin *S *of the ground state changes with *L*, as shown in Figures 1 and 2. For two electrons, *S *= 1 (*S *= 0) when *L *is even (odd). In the case of three electrons, *S *= 3/2 if *L *is a multiple of 3, *S *= 1/2 otherwise. This is explained by the *N*-fold rotational symmetry of the electron configuration in the Wigner molecule [9].

### Electron-hole complex and optical spectrum

Next, we investigate electron-hole complexes: exciton, trion, and biexciton. We fix *R*/*a*_B _= 1. Since the hole motion is almost frozen due to the strong confinement in the quantum dot, the Φ dependence of the ground state of exciton is qualitatively the same as that of an electron confined in *V*_e_(*r*). In the same manner, the Φ dependence of the ground state of trion and biexciton mimics that of two electrons [10]. In particular, two electrons in trion or biexciton form a Wigner molecule, and the period of AB oscillation in the ground-state energy becomes about *h*/2*e *as a function of Φ for trion and biexciton [10].

**Figure 4:**
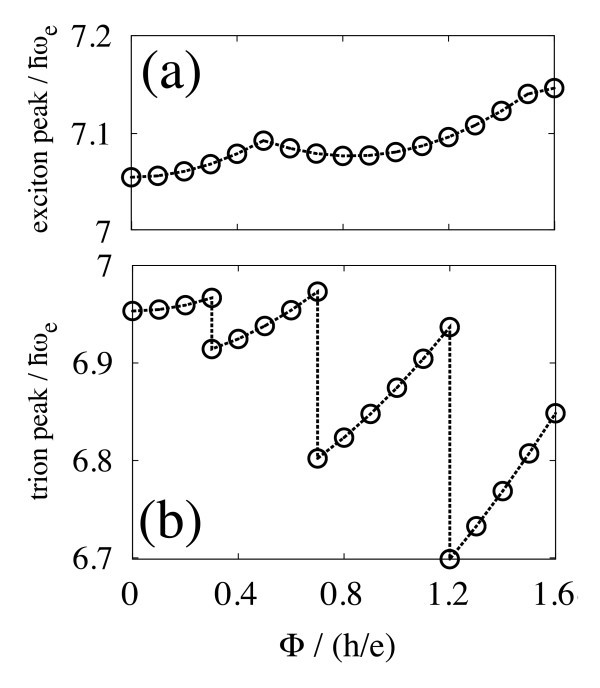
**The luminescence peaks from (a) exciton and (b) trion in the type-II semiconductor quantum dot, as a function of the magnetic flux Φ**. The trion peak suddenly drops as Φ increases by *h*/2*e*.

We examine recombination phenomena. Figure 4 shows the Φ dependence of the luminescence peak from (a) exciton and (b) trion. The behavior of the biexciton peak is qualitatively the same as in Figure 4b. The exciton peak oscillates by the period of about *h*/*e*. On the other hand, the trion peak increases with an increase in Φ and suddenly drops by the period of about *h*/2*e*. The fractional period of *h*/2*e *comes from the period of AB oscillation in the ground state of trion. The discontinuous change is explained by a selection rule for the recombination: The optical transition conserves the orbital angular momentum in two-dimensional systems [11]. A trion with the angular momentum *L *has to decay into an electron with the same angular momentum. As a result, both of the initial and final states of the recombination change at the transition of the trion state. In the case of exciton recombination, the final state is always the vacuum state of the quantum dot, and the peak position is continuous as a function of Φ, as seen in Figure 4a (The recombination of exciton with the angular momentum *L *≠ 0 is forbidden by a selection rule. After the first transition of the electronic state at Φ ≄ *h*/2*e*, excitons get dark in our model. However the forbidden transitions were observed in experiments. This should be ascribed to the disorder of samples which breaks the selection rule.)

**Figure 5:**
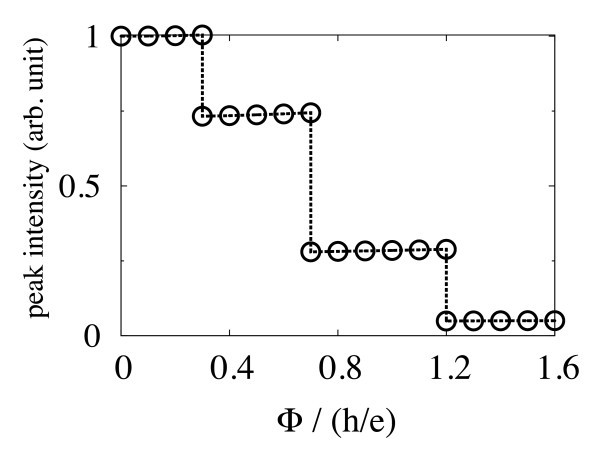
**The intensity of the trion luminescence peak as a function of the magnetic flux Φ**. Reflecting the two-electron wave function, the intensity decreases discontinuously as Φ increases by *h*/2*e*.

Figure 5 shows the intensity of the trion peak as a function of Φ. The intensity of the biexciton peak is approximately the same. The intensity decreases discontinuously at the transition of the electronic state, and approximately takes a constant value until the next transition occurs. Roughly speaking, the height of the intensity plateaus indicates a ratio of 4:3:1:0. The intensity reflects properties of the two-electron wavefunction. This is in good agreement with our theory based on the Heitler-London approximation, in which the correlation effect between electrons is taken into account by a linear combination of two Slater determinants [10].

## Conclusions

We have examined the optical AB effect on trion and biexciton in the type-II semiconductor quantum dots. We have found that two electrons in trion and biexciton form a Wigner molecule. As a result, the ground-state energy oscillates as a function of the magnetic flux by the period of about *h*/2*e*. We have shown that the luminescence spectra from them change discontinuously as the magnetic flux increases by about *h*/2*e*. This indicates the possible observation of Wigner molecules by the optical experiment.

We note that the discontinuous change in the luminescence peaks and intensity stems from the selection rule, which is broken in the presence of disorder. By the selection rule, excitons with the angular momentum *L *≠ 0 should be dark. However, transitions from excitons with finite *L *were observed by experiments in both ZnSeTe and SiGe [4,5]. Possibly, the sudden change of the luminescence spectra would be smeared in such systems. However, the fractional period of *h*/2*e *is a ground-state property and hence, it is expected to be observed even in dirty samples.

## Abbreviations

AB: Aharonov-Bohm.

## Competing interests

The authors declare that they have no competing interests.

## Authors' contributions

RO developed the numerical model, ran the simulation and acquired data. The interpretation of data has been carried out together with RO and ME. ME and HH conceived of the study and participated in its design and coordination.
